# The value of neurologic and cardiologic assessment in breath holding spells

**DOI:** 10.12669/pjms.301.4204

**Published:** 2014

**Authors:** Unsal Yilmaz, Onder Doksoz, Tanju Celik, Gulcin Akinci, Timur Mese, Tuba Sevim Yilmaz

**Affiliations:** 1 Unsal Yilmaz, MD; Department of Pediatric Neurology, Dr. Behcet Uz Children’s Hospital, Izmir, Turkey.; 2 Onder Doksoz, MD; Department of Pediatric Cardiology, Dr. Behcet Uz Children’s Hospital, Izmir, Turkey.; 3 Tanju Celik , MD; Department of Pediatrics, Dr. Behcet Uz Children’s Hospital, Izmir, Turkey.; 4 Gulcin Akinci, MD; Department of Pediatric Neurology, Dr. Behcet Uz Children’s Hospital, Izmir, Turkey.; 5 Timur Mese, PhD. Department of Pediatric Cardiology, Dr. Behcet Uz Children’s Hospital, Izmir, Turkey.; 6 Tuba Sevim Yilmaz, MD; Department of Public Health, Dokuz Eylul University Hospital, Izmir, Turkey.

**Keywords:** Breath-holding spell, Corrected QT, Electroencephalography, Iron deficiency anemia

## Abstract

***Objective*** *:* To evaluate the value of neurologic and cardiologic assessment and also the frequency of iron deficiency anemia in children with Breath Holding Spells (BHS).

***Methods*** *:* The hospital charts of patients diagnosed with BHS between 2011 and 2013 were reviewed retrospectively.

***Results*** *:* A total of 165 children (90 boys, 75 girls) with BHS comprised the study group. A matched group of 200 children with febrile convulsions served as controls. Among the first-degree relatives, 13.3% had BHS, 1.8% had febrile convulsions and 12.1% had epilepsy. The spells were cyanotic in 140 (84.8%) children and pallid or mixed in the remainder. BNS type was simple in 46.7% of patients and complicated in the remainder. Eighteen patients had abnormalities in electroencephalography, however only one patient was diagnosed with epilepsy. Sixty nine (47.9%) patients were found to have iron deficiency anemia.

***Conclusion*** *:* Referral of children with clinically definite BHS to pediatric neurology or pediatric cardiology clinics and performance of echocardiography and EEG investigations for exclusion of heart disease or epilepsy appear unnecessary. However, performance of an electrocardiogram to search for prolonged QT syndrome should be considered although no patient in our series had any cardiologic abnormalities.

## INTRODUCTION

Breath-holding spells (BHS) are among the common benign paroxysmal non-epileptic disorders occurring in healthy otherwise normal children.^1^ The prevalence has been estimated between 0.1% and 4.6% in the general population.^2^^,^^3^

The diagnosis is usually made by description or observation of typical attacks which are characterized by a sequence of clinical events, beginning with a provocation such as minor trauma or emotional upset, followed by a noiseless state of expiration accompanied by color change in the skin as paleness or cyanosis, and finally loss of consciousness and postural tone. Two types are recognized clinically as pallid and cyanotic on the basis of skin color change observed during spells, the former being more common.^4^ Although BHS are often very stressful for the parents observing an episode, these attacks are mostly self limited, and spontaneous resolution without sequelae is anticipated in nearly all cases by school age.^4^

On the other hand, these spells may rarely be an initial symptom of long QT syndromes or paroxysmal cardiac rhythm abnormalities in some patients.^5^ Therefore performance of an electrocardiogram to evaluate for prolonged QT syndrome is strongly considered. Severe pallid spells and less commonly, cyanotic spells may be complicated by myoclonic jerks and generalized seizures, following the crying period.^6^ Iron deficiency anemia (IDA) may be a factor contributing to BHS by reducing the oxygenation of CNS.^7^ Correction of iron deficiency may eliminate these spells.^7^^,^^8^

The aim of the present study was to evaluate the value of neurologic and cardiologic assessment and also to determine the frequency of iron deficiency anemia in children with BHS in daily clinical practice. 

## METHODS

A total of 166 children diagnosed with BHS at the child neurology out-patient clinics at Dr. Behcet Uz Children’s Hospital between 2011 and 2013 were included into the study. Children with a primary neurologic, cardiac or hematologic disease were excluded. The study protocol was approved by the ethical committee of the hospital. Details of the children with BHS and controls are presented in Table-I.

Data from these files were collected, including information on age at admission and at onset of symptoms, sex, perinatal problems (i.e. asphyxia, hypoglycemia, infection, and respiratory distress), developmental history, blood tests, electroencephalography (EEG), electrocardiography (ECG) and echocardiography results and family history among first degree relatives regarding BHS, epilepsy and febrile convulsions. The spells were classified into cyanotic, pallid or mixed types according to the skin color change during episodes. Data on BHS included the type, severity and frequency of attacks, and total number of spells. Two hundred children with a diagnosis of simple febrile convulsions in the similar age and sex group followed-up in the same child neurology out-patient clinic served as controls. 

Hemoglobin concentration, mean corpuscular volume (MCV), serum iron (SI), and total iron binding capacity (TIBC) were measured at the time of the diagnosis. Electrocardiography and echocardiography in all children and EEG in selected cases were carried out for exclusion of organic disease. The QT interval was accepted as the interval between the beginning of the QRS complex and the end of the T wave. The Bazett formula^9^ was used for calculation of corrected QT (QTc), which was recorded in milliseconds (ms), or on a normal–abnormal scale on the charts of the patients. The abnormalities on EEG were divided into background abnormalities without epileptic activity and epileptic abnormalities when it is clearly epileptic. 

In order to study the relation of BHS with IDA, complete blood count and SI, TIBC, and ferritin levels were measured at the time of the diagnosis. The diagnosis of IDA was made according to the age-specific criteria for the diagnosis of anemia.^10^ Patients with IDA were treated with ferrous sulphate solution 6 mg/kg/day orally for three months.

***Statistics: ***All data were descriptively analysed with SPSS 20.0, Chicago, IL, USA. For qualitative data, Pearson Chi-square test was employed. For comparison of quantitative data of two groups, Student’s* t* test and Mann-Whitney U-tests were used for parametric and nonparametric data respectively. P <0.05 values were considered significant.

## RESULTS

In one child with a typical BHS history and a normal initial EEG, the frequency and severity of spells increased over time and the attacks began to occur without any provocation. After obtaining a second EEG which revealed clearly epileptic activity, a diagnosis of epilepsy was made and the patient was excluded from the rest of the analysis. A total of 165 children with BHS and 200 children with febrile convulsions as a control group were studied. Demographic and clinical characteristics of the patients and controls are shown in Table-I and characteristics of BHS are presented in Table-II. 

Results of EEG, ECHO and EEC investigations of children with clinical diagnosis of BHS are shown in Table-III. Eighteen patients (25.7%) had EEG abnormalities without epileptic activity and 4 (5.7) patients had EEG abnormalities with epileptic activity. Echocardiography revealed patent foramen ovale in two patients, pulmonary stenosis in one patient and mitral valve prolapsus in one patient. Electrocardiography was normal in all patients. Corrected QT intervals were recorded in msec in 62 patients, and the mean QTc interval was 397±19.8 msec. In the remainder, QTc interval was recorded as normal. Mean QTc interval was not significantly different between patients with cyanotic and pallid BHS, between the patients with simple and complicated BHS, and also between the patients with and without iron deficiency anemia.

Hematologic data of the patients with breath holding spells and controls are presented in Table-IV. Mean hemoglobin concentrations was significantly lower in the patients with BHS as compared to the controls (11.08±1.20 g/dl in BHS group, 11.53±0.99 g/dI in controls, p = 0.001). Similarly MCV was significantly lower in children with BHS as compared to controls (75.7±7.6 in patients with BHS, 79.2±6.5 in controls, p < 0.001). Sixty (41.4%) patients had low hemoglobin values, 46 (31.7%) patients had low MCV values, 32 (27.8%) patients had low SI values, and 11 patients had low ferritin values compared to normal reference values. Iron deficiency anemia was diagnosed in 47.9% of patients with BHS, whereas 29.9% of patients with febrile convulsions had IDA (p= 0.002). When we compared patients with cyanotic and pallid spells, and the patients with simple and complicated BHS, there was no statistically significant difference in terms of Hb, MCV, iron, TIBC, and ferritin values between the groups (Table-V). 

## DISCUSSION

Breath-holding spells represent an age-limited disorder. It usually begin between the ages of 6 and 24 months of life, peaking in frequency by around 2 to 3 years, and 90% or more of patients have their initial spells by age 2 years.^4^^,^^6^^,^^11^^,^^12^ It may begin as early as during neonatal period,^13^^,^^14^ and almost never after the age of 5 years.^2^ About half of the children stops experiencing spells by age 4 years, and almost all by age 6 years,^4^ beyond which their occurrence is extremely uncommon.^8^^,^^15^ In accordance with previous reports, we found that the mean age of occurrence of BHS was 10.6 months in our series. 

**Table-I:** Demographic and clinical characteristics for children with breath holding spells and controls with febrile convulsions

*Parameters*	*BHS* *n = 165, (%)*	*Controls* *n = 200, (%)*	*p value*
Sex			
Female	75 (45.5)	83 (41.5)	0.448
Male	90 (54.5)	117 (58.5)	
Median Age at onset, months	9	14	
Mean Age at onset, months, ±S.D., (range)	10.62±9.69 (0-50)	11.97±4.24 (2-29)	0.099
<6 month	64 (38.8)	17 (8.5)	
7-12 months	51 (30.9)	101 (50.5)	
12-24 months	39 (23.6)	81 (40.5)	
>24 months	11 (6.7)	1 (0.5)	
Median Age at diagnosis, months	17	20	
Age at diagnosis, months, ±S.D., (range)	21.30±14.69 (1-68)	21.62±10.55 (3-60)	0.817
<6 month	17 (10.3)	6 (3.0)	0.014
7-12 months	26 (15.8)	26 (13.0)	
12-24 months	70 (42.4)	108 (54.0)	
>24 months	52 (31.5)	60 (30.0)	
Mean interval to diagnosis			
Disease duration, months, ±S.D., (range)	11.28±14.25 (1-90)		
Perinatal problems 165	16 (9.7)	24 (12.2)	0.452
Prematurity	7 (4.2)	8 (4.1)	0.750
Perinatal asphyxia	7 (4.2)	11 (5.6)	
SGA	2 (1.2)	5 (2.5)	
Neonatal convulsions	1 (0.6)	0	0.274
Delayed psychomotor development	2 (1.2)	2 (1.0)	0.858
Parental consanguinity	22 (13.4)	26 (13.3)	0.967
BHS in first degree relatives	22 (13.3)		
Epilepsy in first degree relatives	20 (12.1)	29 (14.8)	0.460
Febrile convulsion in first degree relatives	3 (1.8)	42 (21.4)	

BHS = breath-holding spells,

S.D. = standard deviation,

SGA = small for gestational age.

**Table-II:** Characteristics of breath holding spells

*Parameters*	*n = 165, (%)*
Duration of spell, seconds (range)	41.20±25.38 (10-180)
Type of BHSs	
Cyanotic	148 (89.7)
Pallid	11 (6.6)
Mixed	6 (3.6)
Severity of spells	
Simple	78 (47.3)
Complicated	87 (52.7)
Frequency of spells	
>30 per month	22 (13.3)
10-30 per month	19 (11.5)
<10 per month	124 (75.2)
BHS = breath-holding spells	

**Table-III:** Results of EEG, echocardiography and electrocardiography in the patients with breath holding spells and a control with febrile convulsions

	*BHS* *n/N* ^a^ *, (%)*	*Controls* *n/N* ^a^ *, (%)*	*p value*
Abnormal electroencephalography	18/70 (25.7)	40/142 (28.2)	0.706
Background abnormalities	14/70 (20.0)	25/142 (17.6)	0.493
Epileptic abnormalities	4/70 (5.7)	15/142 (10.6)	
Abnormal electrocardiography	0/84		
QTc, ms, ±S.D., (range) n = 62	397±19.80 (350-430)		
Abnormal echocardiography	5/97 (5.1)		
Patent foramen ovale	3/97 (3.1)		
Pulmonary stenosis	1/97 (1.0)		
Mitral valve prolapsus	1/97 (1.0)		

BHS = breath-holding spells; ms = millisecond; QTc: corrected QT; S.D. = standard deviation

^a^ Number of patients with outcome variable/total number of patients.

**Table-IV:** Mean values of blood indexes in the patients with breath holding spells and a control group with febrile convulsions

	BHS n/N^a^, (%)	Controls n/N^a^, (%)	p value
Hemoglobin (mg/dl), ±S.D., (range)	11.08±1.20 (6.4-13.6)	11.53±0.99	0.001
Frequency of low Hb, (%)	60/145 (41.4)	32/134 (23.9)	0.002
MCV (fl), ±S.D., (range)	75.74±7.60 (50-89)	79.26±6.50	
Frequency of low MCV, (%)	46/145±31.7	19/134 (14.2)	0.001
Iron (µg/dl), ±S.D., (range)	50.00±28.18 (7-122)		
Frequency of low iron, (%)	32/115 (27.8)		
Total iron binding capacity, ±S.D., (range)	377.5±57.2 (185-506)		
Ferritin (µg/L), ±S.D., (range)	28.06±26.32 (1-149)		
Frequency of low ferritin (%)	11/104 (10.6)		
Iron deficiency anemia (%)	69/144 (47.9)	40/134 (29.9)	0.002
Iron replacement therapy, (%)	106 (64.2)		

BHS = breath-holding spells; S.D. = standard deviation; MCV = mean corpuscular volume

^a^ Number of patients with outcome variable/total number of patients.

**Table-V:** Comparisons of mean values of blood indexes and corrected QT intervals between various groups of patients with breathe holding spells

	*Patients without IDA*	*Patients with IDA*	*p*	*Patients with cyanotic BHS*	*Patients with pallid/mixed BHS*	*p*	*Patients with simple BHS*	*Patients with complicated BHS*	*p*
Hemoglobin (mg/dl), ±S.D.	11.9±0.61 n =76	10.1±0.98 n =69		11.06±1.23 n =130	11.32±0.8 n =15	0.439	11.1±0.94 n =69	11.0±1.39 n =76	0.730
MCV (fl), ±S.D.	79.3±3.73 n =76	71.9±7.89 n =69	<0.001	75±7.20 n =130	78±5.5 n =15	0.880	76.5±6.60 n =69	75.2±7.53 n =79	0.279
Iron, (µg/dl), ±S.D.	57.8±25.3 n =59	40.0±27.8 n =54	0.001	49.4±28.9 n =103	53.1±19.3 n =12	0.426	48.4±24.7 n =53	51.0±30.8 n =52	0.623
Total iron binding capacity, ±S.D.	364±52.5 n =55	389±59.1 n =49	0.025	378±57 n =97	366±45 n =9	0.606	376±54.4 n =49	379±59.4 n =57	0.789
Ferritin, (µg/L), ±S.D.	32.0±28.8 n =60	22.5±21.4 n =43	0.72	27.1±25.7 n =94	34.6±31.1 n =10	0.282	29.4±30.3 n =51	26.4±21.8 n =53	0.568
Iron deficiency anemia (%)				64/130 (49.2)	5/15 (33.3)	0.243	34/69 (47.6)	35/76 (52.4)	0.698
QTc (ms), ±S.D.	397±20.1 n =31	396±19.7 n =26	0.813	397±20 n =53	396±19 n =9	0.831	399±19.3 n =33	395±20.4 n =29	0.422

BHS = breath-holding spells; S.D. = standard deviation; ms = millisecond; QTc: corrected QT; MCV = mean corpuscular volume.

The mean interval between the onset of spells and admittance to physician was 11.2 months. Although statistically not significant, this interval was longer in patients with a family history of BHS. This may be interpreted as awareness of BHS is a benign phenomenon results in relief of parents. In consistent with previous studies^4^^,^^11^^,^^12^ we found a higher incidence of BHS in males compared to females with a ratio of 1.2:1. As reported previously^16^ there were no significant differences in perinatal data including perinatal asphyxia, premature or SGA birth, and neonatal convulsions when comparing the patients with BHS to the control group. 

A genetic causative factor may be responsible for the disease, and autosomal-dominant inheritance is suggested.^8^^,^^13^^,^^15^ A positive family history of BHS has been reported in up to 30% of children with BHS.^4^^,^^6^^,^^13^^,^^16^ In our series, 13.3% of patients had a family history for BHS, which confirm the previously established familial tendency to BHS. We also found a familial tendency of epilepsy in children with BHS, in accordance with a previous study reporting a familial history of epilepsy in 14.6% patients with BHS.^16^

Complicated BHS comprises 15% of all cases.^2^ In our series, 52.7% of cases had complicated BHS. This relatively high rate was likely caused by referral of more severe attacks to pediatric neurology out-patient clinics. Cyanotic BHS have been reported in 54-62% of cases, whereas pallid BHS in 19-22% of cases, and mixed or unclassifiable spells in 19-24% of cases.^2^^,^^4^^,^^6^^,^^17^ In our series, 89.7% of children had cyanotic spells, a proportion higher than previously reported. The frequency of episodes ranges from several times daily to once yearly.^6^ A previous study has reported that mean attack rate was 5 in a month. Similarly, about 75% of patients in our series experienced less than 10 attacks in a month.

Autonomic nervous system dysfunction is thought to be the primary abnormality in the pathophysiology of BHS.^18^ Prolonged expiration which are mediated by effects causes the cyanotic attacks, whereas pallid spells are mediated by autonomic effects. Both sympathetic and parasympathetic stimulation may affect QT interval.^19^^,^^20^ Lengthening of the QT and QTc interval are associated with ventricular arrhythmias and sudden death in some patients with underlying familial prolonged QT syndrome.^21^ Prolonged QT syndrome is a rare, but potentially malignant, cause of anoxic seizure.^22^ A previous study has reported that patients with pallid/mixed attacks had increased QT dispersion compared to patients with cyanotic attacks.^23^ In our series, none of the patients had prolonged QT interval or any other electrocardiography abnormalities. We did not find any difference between the patients with cyanotic and pallid BHS regarding QTc interval either. However we cannot suggest that it is unnecessary to obtain ECG in the assessment of a child with BHS, since previous reports draw attention to the importance of performing an ECG, at least in children with pallid BSH, in order to identify prolonged QT-syndrome or other cardiac arrhythmia.^16^ One of these studies investigating long term prognosis for children with BHS has reported that a patient suddenly died due to ECG-verified Wolf-Parkinson-White block with a short PQ-interval, slightly broadened QRS-complexes and delta waves. ECG had not been performed during the time the patient was first admitted to hospital for BHS.^18^ Therefore; the persistence of spells into late childhood should be accepted as an alarming sign to search an underlying cardiac disorder.

Occasionally, patients may experience a seizure-like activity, a true epilepsy or even status epilepticus following either a cyanotic or pallid BHS.^24^ In a previous study, 95 children with BHS were followed prospectively, and 15 of them were found to have hypoxic convulsions.^6^ In our series, an infant with an initial history of typical cyanotic BHS and a normal EEG, was subsequently diagnosed with epilepsy. In addition, 4 patients had vertex spikes on their interictal EEGs and two of them experienced convulsive seizures following anoxic periods. Iron therapy ablated spells in one patient and decreased considerably in the other. Thus it appears imperative to rule out other causes of loss of consciousness and EEG investigation appears useful in patients with BHS, when the pattern of attacks changes over time in breath-holders.

Parents are under a great degree of emotional stress of the thought of danger of death during the spells.^25^ Physicians; on the other hand, refer these children to pediatric neurology and pediatric cardiology clinics because of the fear of missing an underlying serious cardiac or neurological disorder. However none of the patients in our series had any relevant cardiac pathology and epilepsy diagnosis was made in only one patient who had an initial diagnosis of BHS.

Treatment of BHS consists mostly of providing reassurance to the parents and attempting behavioural modification. Besides iron treatment has been shown to be effective.^7^^,^^8^^,^^26^ An association between anemia and BHS has been demonstrated previously.^7^^,^^8^ It has been demonstrated that children with BHS had significantly lower hemoglobin concentrations than those of controls,^26^ and from 65% to 69% of patients with BHS had IDA.^9^^,^^23^ Similarly, 69 (47.9%) patients had IDA in our series, and Hb and MCV indices were significantly lower when compared with controls with febrile convulsions.

In conclusion, referral of children with a clinical diagnosis of BHS to pediatric neurology or cardiology clinics is unnecessary, and routine electroencephalography and echocardiography are not appropriate investigations in initial evaluation of patients with BHS.

## Authors Contributions:

Unsal Yilmaz, Tuba Sevim Yilmaz, Tanju Celik, Onder Doksoz, Gulcin Akinci had primary responsibility for protocol development, patient screening, enrolment, data analysis and writing the manuscript. Timur Mese supervised the design and execution of the study, performed the final data analyses. 
